# A Belief Network Reasoning Framework for Fault Localization in Communication Networks

**DOI:** 10.3390/s20236950

**Published:** 2020-12-05

**Authors:** Rongyu Liang, Feng Liu, Jie Liu

**Affiliations:** 1 School of Computer and Information Technology, Beijing Jiaotong University, Beijing 100044, China; liangrongyu@bjtu.edu.cn; 2 Computer Science Department, Computer Science Division, Western Oregon University, Monmouth, OR 97361, USA; liuj@wou.edu

**Keywords:** fault localization, belief networks, message propagation, fault inference, communication networks

## Abstract

A small fault in a large communication network may cause abrupt and large alarms, making the localization of the root cause of failure a difficult task. Traditionally, fault localization is carried out by an operator who uses alarms in alarm lists; however, fault localization process complexity needs to be addressed using more autonomous and intelligent approaches. Here, we present an overall framework that uses a message propagation mechanism of belief networks to address fault localization problems in communication networks. The proposed framework allows for knowledge storage, inference, and message transmission, and can identify a fault’s root cause in an event-driven manner to improve the automation of the fault localization process. Avoiding the computational complexity of traditional Bayesian networks, we perform fault inference in polytrees with a noisy OR-gate model (PTNORgate), which can reduce computational complexity. We also offer a solution to store parameters in a network parameter table, similar to a routing table in communication networks, with the aim of facilitating the development of the algorithm. Case studies and a performance evaluation show that the solution is suitable for fault localization in communication networks in terms of speed and reliability.

## 1. Introduction

In large enterprises, communication networks have become a fundamental infrastructure. Increasingly diverse applications, such as online electronic transactions, network synergetic work, high-security remote monitoring, and even mission-critical remote control and emergency call services all run on top of the networks [1]. Networks are increasing in size and complexity and are moving toward heterogeneity. In such a network, maintaining a higher level of performance and reliability is both a significant task and a challenging problem for fault management. Fault localization is the core component in network fault management. Its purpose is to quickly and accurately locate the root cause of the fault. A good fault localization scheme will reduce network maintenance time and improve the availability of network services [2]. Furthermore, the future network will be more intelligent and adaptive than the currents ones. Therefore, their fault localization methods and techniques need to emphasize the following objectives: automation, accuracy, speed and reliability.

In communication networks, a single fault in one component can produce inconsistent outputs. These abnormal outputs may serve as inputs to other healthy parts of the networks [3]. This phenomenon often causes cascaded faults in a communication network and may cause a large number of alarms to be raised. These abrupt and large alarms are raised in a short period, which can overwhelm even experienced operators [4]. In this case, operators cannot handle every alarm properly but can only acknowledge these alarms. As a consequence, the root causes of alarms or critical alarms may be overlooked, and serious negative consequences would arise due to the lack of responses to these alarms [5]. The network will get worse and worse and lead to the disruption of the communication services. Worse of all, this may cause a catastrophic consequence.

Faults in communication are inevitable and are the root cause of problems [6]. A fault may cause the hardware or software to lose its predefined functions and fail to perform its expected missions. A fault occurring in one network entity may affect another network entity in performing its original functions—e.g., the faults of a switch fan may cause the mainboard to suspend due to overheating. As a result, these faults can form a directed acyclic propagation graph. Alarms are the external symptoms of the faults and can be observed by network operators, but, in general, faults cannot be directly observed. In practical applications, we frequently observe the phenomenon that the alarms arise one by one. In essence, the causal relationship is not between alarms, but rather between faults [7]. Therefore, our objective was to localize the root cause of faults using the observed large number of alarms.

Aiming at this purpose, wide and profound research has been conducted over the past few decades. Fault localization has been achieved by many existing approaches and techniques, such as model-based approaches, rule-based approaches, case-based approaches, and emerging machine learning techniques. However, graceful schemes that represent either the causal relationship between the network events or the dependency relationship between the network entities are lacking. The absence of a correct causal inference model in these methods causes them to fail in instances of novel problems [8,9]. There are also few works that consider an overall framework for knowledge storage, inference, and message transmission in fault localization. In [10], Judea Pearl introduced a belief updating mechanism in belief networks. In this mechanism, each node can receive and send messages from its neighboring nodes, and then calculate its own belief based on these received messages. This message propagation mechanism provides a theoretical foundation to construct an overall framework for fault localization.

Motivated by the belief propagation mechanism proposed by Judea Pearl in [10], we present an application of belief networks using the message propagation mechanism for fault localization in a communication network, called polytree with noisy OR-gate (PTNORgate). Based on this mechanism, we propose an overall framework, which allows for knowledge storage, inference, and message transmission. We also offer a solution to store parameters in a network parameter table, such as a routing table in communication networks, with the aim of facilitating the development of the algorithm. To reduce the computational complexity in the traditional Bayesian network, we propose an improvement by performing the fault inference in PTNORgate, which allows a root cause inference in polynomial time. Our schemes are almost automatic and can perform the reasoning process in an event-driven manner.

The main contributions of this paper are summarized as follows:

1. We propose an overall framework to perform fault localization in communication networks, which allows for knowledge storage, inference, and message transmission.
2. We apply PTNORgate to address the computational complexity problem. This helps to avoid the computational complexity in the calculation process of fault reasoning.
3. We offer a solution for storing parameters in a network parameter table, such as a routing table in communication networks, with the aim of facilitating the development of the algorithm.
4. The scheme that we offer carries out the reasoning process in an event-driven manner. This scheme improves the degree of automation of the localization process and reduces human intervention.

The remainder of the paper is organized as follows. We review the related work on fault localization in Section 2. In Section 3, we introduce the concepts and benefits of belief networks for fault localization, and we present our framework and techniques for fault localization in Section 4. A fault scenario of the transmission network is studied in Section 5. We carried out a performance evaluation, and the discussion of the results is provided in Section 6. We provide a conclusion in Section 7.

## 2. Related Works

A consolidated taxonomy on various approaches and techniques for fault localization in computer networks has been presented in [6]. Generally, these approaches and techniques are broadly categorized as model-based approaches, rule-based approaches, case-based approaches, and emerging machine learning techniques. They aim to make fault diagnosis intelligent and automated. Some of the most preeminent examples will be briefly presented below.

Model-based approaches describe the behavior of the system as a mathematical model by means of expert knowledge. A profound understanding of the underlying structure and operating mechanism of the system is required [11,12]. In [13], a simple network management protocol (SNMP) based on a management model is proposed. The model can localize the root cause of the event and give advice to operators for solving problems; however, these models may be difficult to obtain and keep up to date.

In [14], a rule-based approach is proposed for communication network operation and management. Such a model generally consists of three parts: a rule base, a rule discovery engine, and an inference engine. The first two parts can be achieved by iterative and incremental algorithms. New rules are constantly added into the rule base by performing iteration algorithms in different conditions. The inference engine determines which rule is the most satisfied with the given situation [15]. Updating and enriching the knowledge base and carrying out the inference process are all more complex. Especially in a network in which the topology frequently changes, a large number of rules needs to be updated frequently. Therefore, this method is not well suited for such a network, although many emerging techniques have been proposed to automatically learn rules based on observed symptoms [16,17].

Case-based approaches rely on human experience obtained from the past fault cases [18,19,20]. A new experience is stored in the case base when a problem has been solved. The new experience would be retrieved and reused for future problems. In [2], the authors presented a hybrid approach that combines case-based reasoning and Bayesian networks (CBR-BN) to identify the root cause of faults. When a fault occurs, the approach carries out fault localization as follows: (1) the fault is viewed as a problem-case; (2) the existing case is matched in the case base; (3) if there is a similar case, then that solution case is identified and applied; (4) if there are no similar cases, then Bayesian inference is carried out, and a new outcome is obtained; (5) finally, the outcome is saved to the case base as a new solution case and reused for future problems. Similar to the rule-based techniques, the main limitations of the case-base techniques come from the time required to update a large number of cases, match a case, and enrich its case base.

In [21], the authors utilize machine learning-based techniques for fault identification and localization in the communication network. The model takes into account the packet loss, end-to-end delay, and aggregate flow rate captured from the networks in normal working states and different fault scenarios. In [22], the authors proposed a solution that uses deep learning to deal with the link handover fault of 5G networks when the mobile device moves from one base station to another base station. Machine learning-based techniques and approaches are well known as a powerful solution for fault localization for complex communication networks [23,24,25,26]. These solutions require a long training period and a large amount of sample data in fault scenarios to train their learning models. Such work is not always feasible with high-reliability and high-security networks. In addition, these solutions lack correct causal representation and lack interpretation for results.

Although the various fault location methods have been widely used over the past few decades, we need a method that can deal with the complex causal relations between failures and symptoms. Several works propose the use of the belief network model. A belief network provides an intuitive representation of causal relationships, and can imitate human thinking to perform a series of reasoning tasks.

A Bayesian network is a directed acyclic graph (DAG). According to [2,27,28,29], networks are considered the most powerful fault diagnosis techniques and have been widely used in various fields, such as mine seismic event discrimination [30,31], and some works considering intrusion detection in wireless communication networks, which could also be helpful in fault localization [32]. Bayesian network techniques are also widely used in mechanical equipment [33,34,35], electronic equipment [36,37], thermal power plants [38,39], petrochemical plants [40,41], nuclear power plants [42,43], and medical diagnoses [44,45,46].

Until now, Bayesian networks have attracted increasing attention in the fault localization field because they can represent the complex causal relationships between faults and symptoms and make causality inferences among them. Few works deal with the overall framework for fault localization in communication networks. In this vein, we propose an overall framework for fault localization in communication networks, which allows for knowledge storage, inference, and message transmission. We also offer a solution to store parameters in a network parameter table, such as routing tables in communication networks, with the aim of facilitating the development of the algorithm. In particular, the scheme carries out the reasoning process in an event-driven manner. This manner improves the degree of automation of the localization process and reduces human intervention.

## 3. Belief Networks as a Fault Propagation Method

Here, we propose applying a message propagation mechanism of belief networks to address fault localization problems in communication networks. In this schema, the impact of each event propagates through the network between neighboring nodes in a message-passing manner. This modeling schema exposes either the dependencies among the network entities or the causal relationship among events [29]. Relying on these mechanisms of the belief networks, the fault propagation models have attracted increasing attention and have been widely used in various systems for fault localization.

In a belief network, we view each node not merely as a variable that represents an event but also as a separate processor that maintains the network parameters (prior probability, posterior probability, and conditional probability). The belief network provides an overall framework for storing knowledge, transferring messages, and carrying out causal reasoning. When an event occurs, each node in the belief network will exchange messages with its neighboring nodes; that is, it updates its own beliefs by receiving messages from its neighbors and sends new beliefs to its neighboring nodes. This state will continue until the events disappear or a new equilibrium is reached in the network. In the new equilibrium state, each node will be reassigned a new probability value. The higher the probability value, the more probable the fault’s root cause. Therefore, fault localization problems may be translated into probability calculation problems.

The belief network is a directed acyclic graph. A graphical representation of the belief network has many advantages in modeling the fault propagation model, as stated in [47]. First, the representation of this structure is transparent for causal reasoning, and it exposes information about the structure so that we can easily understand its semantics. In contrast, an opaque reasoning model easily gives us an unexplained or even undesirable answer; second, the graphical model represents a perceivable dependent relationship that can be used effectively for causal reasoning; third, this structure facilitates the development of a viable model of human reasoning. Whether the models produced by this structure are from human expert knowledge or by learning from data, they always provide a good approximation to human thinking. Much more surprising is the fact that they can sometimes reveal the hidden information in the networks and offer novel insight into the network system’s underbellies.

### 3.1. The Definition and Notations of Belief Networks

Depending on the application in the communication networks, belief networks can be defined as follows.

The belief network is a directed acyclic graph (DAG) in which each node represents a $\left\{0,1\right\}$-value random variable. The directed edges that link between two nodes represent an existence of a causal relationship between two variables. Strengths of the influence of these causal relationships are measured by conditional probability. The nodes (random variables) in the belief network are denoted by capital letter *X*, The set of nodes is denoted by *X* = $\left\{X_{1},X_{2},\ldots ,X_{n}\right\}$, where $X_{i}$ indicates the *i*-th node. $X_{i}\to X_{j}$ represents a directed edge between node $X_{i}$ and $X_{j}$, where $X_{j}$ is the child of $X_{i}$, $X_{i}$ is the parent of $X_{j}$. Let $Par\left(X_{i}\right)=\left\{X_{i_{1}},X_{i_{2}},\ldots ,X_{i_{n}}\right\}$ be the set of all parents of $X_{i}$. $P_{i}$ is the conditional probability matrix associated with a random variable $X_{i}$, and $P\left(x_{i},x_{i_{1}},x_{i_{2}},\ldots x_{i_{n}}\right)=P\left(X_{i}=x_{i}|X_{i_{1}}=x_{i_{1}},X_{i_{2}}=x_{i_{2}},\ldots ,X_{i_{n}}=x_{i_{n}}\right)$. An evident *e* is an observed symptom.

Here, the set of nodes *X* is divided into two categories, $X_{F}$ and $X_{A}$, which represent the set of all fault nodes and alarm nodes, respectively. $X=\left(X_{F},X_{A}\right)$, $X_{F}=\left\{X_{F_{1}},X_{F_{2}},\ldots ,X_{F_{n}}\right\}$ and $X_{A}=\left\{X_{A_{1}},X_{A_{2}},\ldots ,X_{A_{n}}\right\}$. The state value of node $X_{F_{i}}\left(X_{A_{i}}\right)$ is 0 or 1, which represents the *i*-th fault (alarm) is absent (inhibitory) or present (active), respectively. If the state value of a node is 1, we say that the variable is instantiated.

### 3.2. The Noisy OR-Gate Model

It should be pointed out that these inference processes cannot avoid the exponential blowup with the number of nodes in general belief networks. The probability calculation is NP hard [47]. To overcome this limitation, we are proposing a simplified reasoning model called noisy OR-gate to reduce the complexity of the belief network’s inference process, while retaining the advantages of the belief network’s inference technique. As a result, the reasoning model shows a good performance in terms of speed, accuracy, and automation.

**Figure 1:**
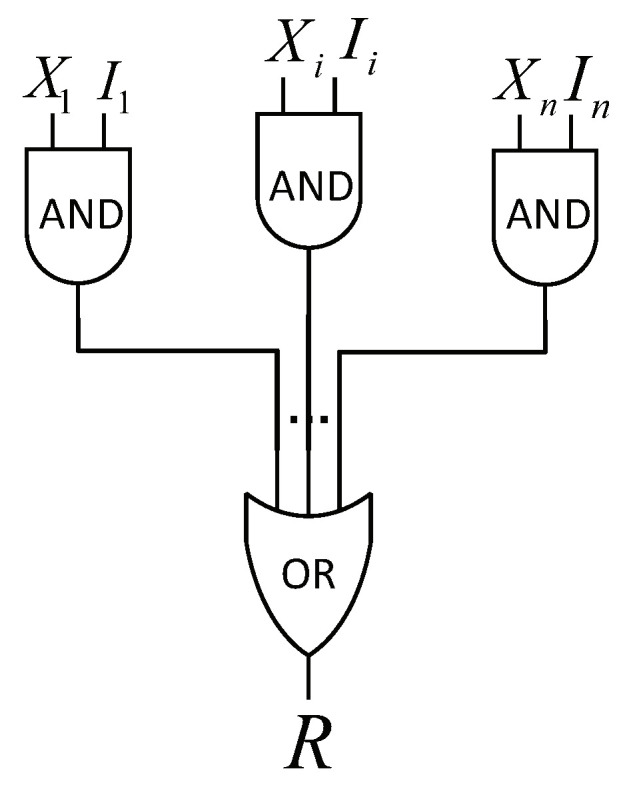
The noisy OR-gate model.

Each variable in the simplified noisy OR-gate model is a binary-valued variable in the belief network. Each variable consists of a causal factor and an inhibitory factor, as shown in Figure 1. The event *R* represents a consequent or prediction of the input. The input $X=\left(X_{1},X_{2},\ldots X_{n}\right)$ represents explanations or conditions that may result in the occurrence of *R*. $I=\left(I_{1},I_{2},\ldots ,I_{n}\right)$ represents inhibitors that can prevent the occurrence of *R*.

In the noisy OR-gate model [10], we assume that all potential causes of the same consequent are independent. This assumption of independence is suitable for investigating probabilistic fault localization techniques and is ubiquitous in the area of fault localization. Instead of a conditional probability matrix of traditional belief networks, the noisy OR-gate model lets each alternative cause separately hold the weight associated with each of its likely consequences. In the reasoning process, the consequent or prediction is absent only if all inhibitors associated with each of the likely causes are activated. In other words, if the consequent or prediction is present, at least one inhibitor associated with the present cause remains inactive. In a set of conditions, one may cause some specific event, when several of these conditions occur simultaneously, the occurrence probability of the event does not diminish. For example, there are many potential reasons, such as network congestion, failed connection, or a destroyed forwarding table. Each of them may individually cause a service disruption in a communication network. When a communication network suffers from several of these causal factors simultaneously, the occurrence probability of service disruption will only be higher. The most surprising aspect of these refinements is that it does not need to store the conditional probability matrix. It is guaranteed to perform reasoning tasks in polynomial time.

## 4. Fault Localization Techniques

Here, the noisy OR-gate model performs causal reasoning tasks in polytrees. The polytree is a singly connected network with no more than one path between any two nodes. This structure helps to avoid the loops in the networks and facilitates the development of the fault propagation algorithm.

### 4.1. Messages Fuse and Propagate in Belief Networks

**Figure 2:**
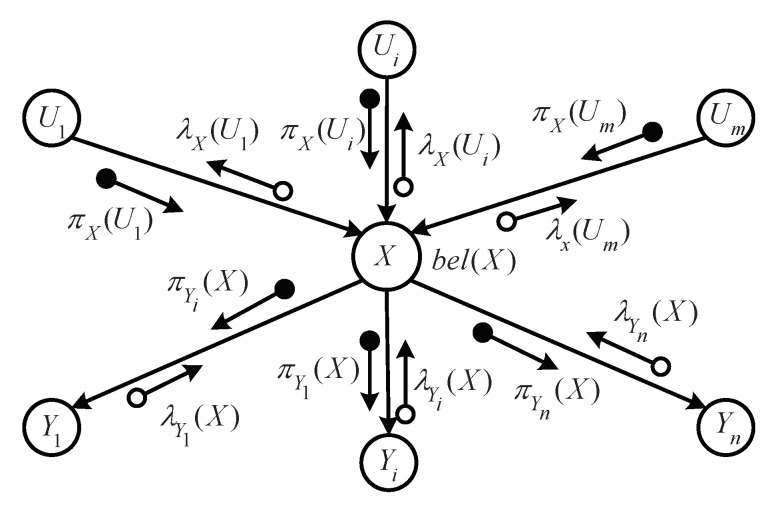
Message propagate in Pearl’s belief propagation network. Nodes $\left(U_{1},U_{2},\ldots ,U_{m}\right)$ and $\left(Y_{1},Y_{2},\ldots ,Y_{n}\right)$ are node $X^{\prime }s$ parents and children, respectively.

The noisy OR-gate model utilizes a message-passing mechanism to exchange messages in belief networks, as shown in Figure 2. Each node exchanges messages with its neighboring nodes in the reasoning process [10,48]. Initially, the network is in a stable state, no event occurs, and all nodes remain in their waiting state until messages are received. As soon as events arise in the network, the nodes associated with events are activated. The influences produced by activated nodes are spread to their neighboring nodes along the edges between them. Each node then (1) receives all messages from their neighboring nodes; (2) absorbs and produces new messages by the belief update algorithm that we introduce in Section 4.2; (3) sends these new messages to their neighboring nodes. This process continues until the abnormal events are removed or a new equilibrium is reached in the belief networks. Both the processes of absorbing and producing messages are detailed in Section 4.2 and Section 4.3, respectively. In this fashion, we can track the changing environment in the network and provide a coherent interpretation.

As shown in Figure 2, we consider a general node *X*, which excludes the root nodes and leaf nodes in the belief networks. The set of nodes $\left(U_{1},U_{2},\ldots ,U_{m}\right)$ and $\left(Y_{1},Y_{2},\ldots ,Y_{n}\right)$ are node $X^{\prime }s$ parents and children, respectively. Node *X* received $\left(\pi_{X}\left(U_{1}\right),\pi_{X}\left(U_{2}\right),\ldots ,\pi_{X}\left(U_{m}\right),\right)$ and $\left(\lambda_{Y_{1}}\left(X\right),\lambda_{Y_{2}}\left(X\right),\ldots ,\lambda_{Y_{n}}\left(X\right),\right)$ messages from its parents and children, respectively, and sends $\left(\lambda_{X}\left(U_{1}\right),\lambda_{X}\left(U_{2}\right),\ldots ,\lambda_{X}\left(U_{m}\right)\right)$ and $\left(\pi_{Y_{1}}\left(X\right),\pi_{Y_{2}}\left(X\right),\ldots ,\pi_{Y_{n}}\left(X\right)\right)$ to its parents and children, respectively. Node *X* triggers the calculation mechanism to update its own belief via collected messages. It should be pointed out that the belief update can be carried out gradually, and need not be interrupted until all the information is collected.

### 4.2. The Belief Update in Belief Networks

In this section, we introduce the belief update process of nodes in the belief networks based on the message propagation mechanism. Each node receives $\pi \left(X\right)$ and $\lambda \left(X\right)$ messages from its parents and children, respectively. As shown in Figure 2, we can obtain $\pi \left(X\right)$ and $\lambda \left(X\right)$ in node *X* as follows:

(1) $$\pi \left(x\right)=\left\{\begin{array}{cc} \pi^{0}\left(x\right)=\alpha \prod_{i=1}^{m}\left(1-c_{i}\pi_{iX}\right) & \mathrm{if}\;x=0\\ \pi^{1}\left(x\right)=\alpha \left[1-\prod_{i=1}^{m}\left(1-c_{i}\pi_{iX}\right)\right] & \mathrm{if}\;x\;=\;1 \end{array}\right.$$

(2) $$\lambda \left(x\right)=\left\{\begin{array}{cc} \lambda^{0}\left(x\right)=\prod_{i=1}^{n}\lambda_{Y_{i}}^{0}\left(x\right) & \mathrm{if}\;x\;=\;0\\ \lambda^{1}\left(x\right)=\prod_{i=1}^{n}\lambda_{Y_{i}}^{1}\left(x\right) & \mathrm{if}\;x\;=\;1 \end{array}\right.$$

Then, node *X* calculates its own new belief bel(x), as follows,

(3) $$bel\left(x\right)=\left\{\begin{array}{cc} \alpha bel^{0}\left(x\right)=\alpha \lambda^{0}\left(x\right)\prod_{i1}^{m}\left(1-c_{i}\pi_{iX}\right) & \mathrm{if}\;x\;=\;0\\ \alpha ble^{1}\left(x\right)=\alpha \lambda^{1}\left(x\right)\left[1-\prod_{i=1}^{m}\left(1-c_{i}\pi_{iX}\right)\right] & \mathrm{if}\;x\;=\;1 \end{array}\right.$$

In the above equations, α is a normalizing constant that renders $\sum_{x}bel\left(x\right)=1$, and $q_{i}$ represents the probability that the *i*-th inhibitor is active, so we denote by $c_{i}=1-q_{i}$ the probability that the *i*-th potential endorses the event X=true. We let $\pi_{iX}$ represent the message that the *i*-th parent $U_{i}$ sends to *X*. $\lambda_{Y_{i}}^{0}\left(x\right)$ represents the inhibited evidence that *X* receives from its *i*-th child, and $\lambda_{Y_{i}}^{1}\left(x\right)$ represents the active evidence that *X* receives from its *i*-th child. $\lambda^{0}\left(x\right)$ represents the inhibited evidence that *X* receives from all of its children, and $\lambda^{1}\left(x\right)$ represents the active evidence that *X* receives from all of its children. x=1 and x=0 represent the presence and absence of events, respectively.

Based on the description above, node *X* receives π(x) and λ(x) messages from its parents and children, respectively. On the other hand, *X* sends λ(x) and π(x) messages to its parents and children, respectively. We denote by $\lambda_{X}\left(u_{i}\right)$ the message that *X* sends to its *i*-th parent, and denote by $\pi_{Y_{i}}\left(x\right)$ the message that *X* sends to its *i*-th child. We calculate them as follows:

(4) $$\lambda_{X}\left(u_{i}\right)=\beta \left[\lambda^{1}\left(x\right)-q_{i}^{u_{i}}\left(\lambda^{1}\left(x\right)-\lambda^{0}\left(x\right)\right)\sum_{k\neq i}\left(1-c_{k}\pi_{kX}\right)\right]\;u_{i}=0,1$$

(5) $$\pi_{Y_{i}}\left(x\right)=\alpha \sum_{k\neq i}\lambda_{Y_{k}}\left(x\right)\pi_{x}\left(u\right)$$

In the above equations, β is any constant. $u_{i}=1$ and $u_{i}=0$ represent the parent $u_{i}$, which is active and inhibited, respectively.

### 4.3. The Storage Mechanism of Belief Networks

As mentioned in the previous section, belief networks have been viewed not merely as a computer architecture but also as a memory for storing knowledge. Similar to the way in which each router in the network maintains a routing table, each node in the belief network maintains a network parameter table. In the parameter table, node X records the information, as shown in Table 1. Node *X* receives $\lambda_{Y_{i}}^{0}$ and $\lambda_{Y_{i}}^{1}$ messages from each of its children by Equation (4). $\lambda^{0}\left(x\right)$ and $\lambda^{1}\left(x\right)$ are calculated by Equation (2). Likewise, Node *X* receives message $\pi_{X}^{0}\left(u_{i}\right)$ from each of its parents by Equation (5), and $\pi^{0}\left(x\right)$ and $\pi^{1}\left(x\right)$ are calculated by Equation (1). p(x) is the prior probability of *X* and the bel(x) is its updated belief. It should be pointed out that node *X* only records the $q_{i}$ incoming from its parents. In addition, a hidden variable $\pi^{1}\left(x\right)$ can be calculated by $\pi^{1}\left(x\right)=1-\pi^{0}\left(x\right)$.

We now find it more convenient to calculate the updated belief bel(x) of each node in the belief networks after every node receives π(x) from each of its parents and λ(x) from each of the children. This data storage solution would have been more useful for understanding and developing the message propagation algorithm. As an example, an application of the belief propagation algorithm to fault localization is given in Section 4.4.

### 4.4. Application of the Belief Propagation Algorithm to Fault Localization

**Figure 3:**
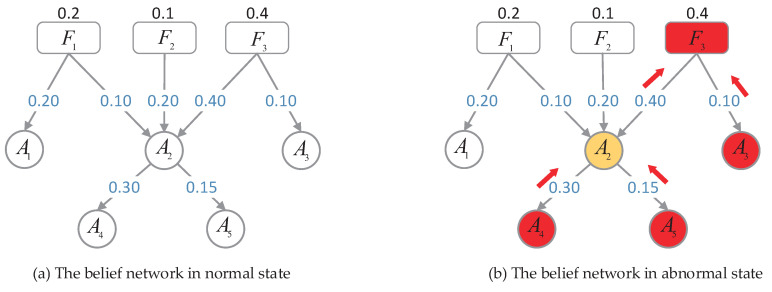
An example of the reasoning process for fault localization. (**a**) Depicts the network is in a normal state; there are no event occurs, and no alarms are raised in the networks. (**b**) Shows the network in an abnormal state. The red circles with a capital letter and number represent alarms that are raised in the network, and the red rounded rectangle with a capital letter and number indicates the fault identified by alarms. The yellow circle with a capital letter and number represents a potential alarm, and the red arrow indicates the direction of causal reasoning.

One example of a belief network model corresponding to a small communication network is depicted in Figure 3. A fault may result in a set of alarms; in the same way, one alarm can also trigger other alarms. Based on the knowledge we have gained from human experience or learned from data using machine learning algorithms, the prior probability of fault is assigned to each corresponding fault node, and the conditional probability that measures the strength of dependency between neighboring nodes is recorded in children nodes.

In the application, the model established the boundary conditions as follows:

- Fault nodes. If node *X* is a fault node, we set π(x) to be equal to its prior probability.
- Leaf nodes. Alarm node *X* is a node with no children. If *X* is instantiated, we set $\lambda^{1}=1,\lambda^{0}=0$. In contrast, if *X* is in a normal state, we set $\lambda^{1}=0,\lambda^{0}=1$. In addition, if *X* has only one parent, in order to prevent the parent from receiving $\lambda^{0}\left(x\right)=0$, the message propagation between *X* and its one parent is restricted to $\left(c_{i},q_{i}\right)$. In other words, we assume that node *U* is the only parent of *X*, and the conditional probability between them is $\left(c_{i},q_{i}\right)$, if *X* is in a normal state, then $\lambda_{x}^{0}\left(u\right)=c_{i}$, $\lambda_{x}^{1}\left(u\right)=q_{i}$. In addition, if *X* is instantiated, $\lambda_{x}^{0}\left(u\right)=q_{i}$, $\lambda_{x}^{1}\left(u\right)=c_{i}$.
- Instantiated node. If node *X* is instantiated, we set bel(x)=1, $\lambda^{1}\left(x\right)=1$ and $\lambda^{0}\left(x\right)=0$ regardless of the other values in the expression. Therefore, node *X* is turned into a leaf node, and the message propagation is a block between *X* and its children.

In order to perform the event-driven fault localization task in the communication network, in our study, we adopted a self-activated message propagation algorithm for fault localization, as follows. The process of the fault localization algorithm starts with any nodes in the belief networks that are instantiated. As stated earlier in the introduction, the status value of the instantiated node is assigned 1, that is bel(x)=1, $\lambda^{1}\left(x\right)=1$, $\lambda^{0}\left(x\right)=0$. The algorithm then starts performing the process of fault localization in an event-driven manner, until events disappear or a new equilibrium is reached in the network. The algorithm is executed by the following procedure:

Step 1. The current belief network is initialized. In this phase, no event occurs, and no evidence arises in the networks. As a result, each node, except for the root nodes, receives π messages from each of its parents, and each node, except for the leaf nodes, receives λ messages from each of its children. Each alarm node is assigned a probability value by Equations (1) and (2).

Step 2. When one node *X* is instantiated, then bel(x)=1, $\lambda^{0}=0$, $\lambda^{1}=1$. The fault localization model starts to perform the fault localization process.

Step 3. The *X* ’s neighboring nodes calculate the new belief bel(x) based on the received messages, and send new π and λ messages to its children and parents, respectively.

Step 4. Step 3 is repeated along the chain until a new equilibrium is reached or the abnormal events have been moved in the networks.

Step 5. A group of faulty nodes are found, and the nodes are arranged in descending order of probability. The higher the probability value, the more probable it is that the alarm occurs.

Step 6. The fault’s root cause is estimated. One fault, or a combination from the set of faults, that provides the best explanation to all present alarms is selected.

**Figure 4:**
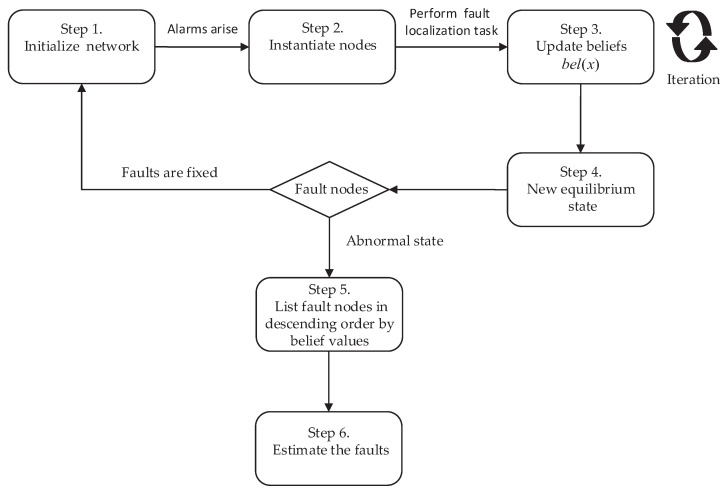
The process of fault localization.

These processes are also detailed in Figure 4.

Figure 3 shows a network with three fault nodes and five alarm nodes. The dependency relationship of the entities in the network was mapped into a polytree. Each network event corresponds to a node in the belief network. For example, network event $alarm_{1}$ corresponds to node $A_{1}$ in the belief network. The set of parameters about the network is labeled in Figure 3.

From Figure 3, we know the prior probability of the fault nodes: $p(F_{1})=0.2$, $p(F_{2})=0.1$ and $p(F_{3})=0.4$. The probability values of the inhibitor from parents to children are $q_{F1A1}=0.20$, $q_{F1A2}=0.10$, $q_{F2A2}=0.20$, $q_{F3A2}=0.40$, $q_{F3A3}=0.10$, $q_{A2A4}=0.30$, and $q_{A2A5}=0.15$. We can obtain the belief distribution of each alarm node by the formula $\left(1-\prod_{i}\left(1-c_{i}\pi_{iX}\right),\prod_{i}\left(1-c_{i}\pi_{iX}\right)\right)$,

$$\pi \left(a_{1}\right)=\left(\pi^{1}\left(a_{1}\right),\pi^{0}\left(a_{1}\right)\right)=\alpha \left(1-\left[1-\left(1-0.20\right)\times 0.2\right],\left(1-0.20\right)\times 0.2\right)=\left(0.16,0.84\right)$$

$$\begin{array}{cc} \; & \pi \left(a_{2}\right)=\left(\pi^{1}\left(a_{2}\right),\pi^{0}\left(a_{2}\right)\right)\\ & \;\;=\alpha (1-[1-(1-0.10)\times 0.2]\times [1-(1-0.20)\times 0.1]\times [1-(1-0.40)\times 0.4],\\ & \;\;[1-(1-0.10)\times 0.2]\times [1-(1-0.20)\times 0.1]\times [1-(1-0.40)\times 0.4])\\ \; & \;\;=(0.4267,0.5733) \end{array}$$

In the same way, we can obtain $\pi \left(a_{3}\right)=\left(0.36,0.64\right)$, $\pi \left(a_{4}\right)=\left(0.2987,0.7013\right)$ and $\pi \left(a_{5}\right)=\left(0.3627,0.6673\right)$.

We assume that $alarm_{3}$, $alarm_{4}$ and $alarm_{5}$ arise in the network, then the nodes $A_{3}$, $A_{4}$ and $A_{5}$ are instantiated in the belief network, where $\lambda^{1}\left(A_{3}\right)=1$, $\lambda^{0}\left(A_{3}\right)=0$; $\lambda^{1}\left(A_{4}\right)=1$, $\lambda^{0}\left(A_{4}\right)=0$ and $\lambda^{1}\left(A_{5}\right)=1$, $\lambda^{0}\left(A_{5}\right)=0$. Because $A_{4}$ and $A_{5}$ are leaf nodes, $A_{2}$ receives $\left(\lambda_{A4}^{1}\left(a_{2}\right)=0.70,\lambda_{A4}^{0}\left(a_{2}\right)=\;0.30\right)$ and $\left(\lambda_{A5}^{1}\left(a_{2}\right)=0.85,\lambda_{A5}^{0}\left(a_{2}\right)=0.15\right)$ messages from $A_{4}$ and $A_{5}$, respectively. $\left(\lambda_{A2}^{1}\left(a_{2}\right)=0.595,\lambda_{A2}^{0}\left(a_{2}\right)=0.045\right)$ is generated by Equation (2). $Fault_{3}$ receives $\left(\lambda_{A_{3}}^{1}\left(a_{2}\right)=0.90,\lambda_{A_{3}}^{0}\left(a_{2}\right)=0.10\right)$ from $A_{3}$. As a result, the new belief distribution of $A_{2}$ can be calculated by Equation (3):

$$\begin{array}{cc} bel\left(a_{2}\right) & =\alpha \left(\lambda^{1}\left(a_{2}\right)\pi^{1}\left(a_{2}\right),\lambda^{0}\left(a_{2}\right)\pi^{0}\left(a_{2}\right)\right)\\ \; & \;\;=\alpha (0.595\times 0.4267,0.045\times 0.5733)\\ \; & \;\;=\alpha (0.2539,0.0258)\\ \; & \;\;=(0.9078,0.0922) \end{array}$$

$Fault_{3}$ receives the following messages from $alarm_{2}$:

$$\begin{array}{cc} \; & \lambda_{A_{2}}^{1}\left(f_{3}\right)=\lambda^{1}\left(a_{2}\right)-q_{F_{3}A_{2}}\left(\lambda^{1}\left(a_{2}\right)-\lambda^{0}\left(a_{2}\right)\right)\left(1-c_{F_{1}A_{2}}\pi_{a_{2}}\left(f_{1}\right)\right)\left(1-c_{F_{2}A_{2}}\pi_{a_{2}}\left(f_{2}\right)\right)\\ \; & \;\;=0.595-0.4\times (0.595-0.045)\times (1-0.9\times 0.2))\times (1-0.8\times 0.1)\\ \; & \;\;=0.4290 \end{array}$$

$$\begin{array}{cc} \; & \lambda_{A_{2}}^{0}\left(f_{3}\right)=\lambda^{1}\left(a_{2}\right)-\left(\lambda^{1}\left(a_{2}\right)-\lambda^{0}\left(a_{2}\right)\right)\left(1-c_{F_{1}A_{2}}\pi_{a_{2}}\left(f_{1}\right)\right)\left(1-c_{F_{2}A_{2}}\pi_{a_{2}}\left(f_{2}\right)\right)\\ \; & \;\;=0.595-(0.595-0.045)\times (1-0.9\times 0.2))\times (1-0.8\times 0.1)\\ \; & \;\;=0.1800 \end{array}$$

Therefore,

$$\begin{array}{cc} \; & \lambda^{1}\left(f_{3}\right)=\lambda_{A_{2}}^{1}\left(f_{3}\right)\lambda_{A_{3}}^{1}\left(f_{3}\right)\\ \; & \;\;=0.4290\times 0.90\\ \; & \;\;=0.1800 \end{array}$$

$$\begin{array}{cc} \; & \lambda^{0}\left(f_{3}\right)=\lambda_{A_{2}}^{0}\left(f_{3}\right)\lambda_{A_{3}}^{0}\left(f_{3}\right)\\ \; & \;\;=0.1800\times 0.1\\ \; & \;\;=0.0180 \end{array}$$

We then obtain the $Fault_{3}$ belief:

$$\begin{array}{cc} \; & bel^{1}\left(f_{3}\right)=\lambda^{1}\left(f_{3}\right)\pi^{1}\left(f_{3}\right)\\ \; & \;\;=0.3861\times 0.4\\ \; & \;\;=0.1544 \end{array}$$

$$\begin{array}{cc} \; & bel^{0}\left(f_{3}\right)=\lambda^{0}\left(f_{3}\right)\pi^{0}\left(f_{3}\right)\\ \; & \;\;=0.0180\times 0.6\\ \; & \;\;=0.0108 \end{array}$$

Finally, the updated belief distribution of $fault_{3}$ is (0.9346, 0.0654).

Each node can calculate its own belief distribution by receiving the messages from its neighbors. Therefore, we obtain the final belief distribution of each variable in the same way, which is shown in Table 2. It is obvious that $alarm_{3}$, $alarm_{4}$ and $alarm_{5}$ are caused by $fault_{3}$.

It can be observed in the above example that the occurrence of $alarm_{4}$ and $alarm_{5}$ is caused by $fault_{3}$. $Alarm_{2}$, with the high occurrence probability without receiving alarm annunciation, can be explained by one of two factors: either the uncertainty of the network leads to alarm loss, or $alarm_{2}$ is not triggered due to its higher threshold value of the performance.

## 5. Case Study

In this section, a typical fault scenario in a communication network is studied. This study is supported by the Chinese government and the research foundation of a railway company. As a case example for applying and verifying the proposed approach, we selected a fault scenario in an optical transport network used in the railway business.

**Figure 5:**
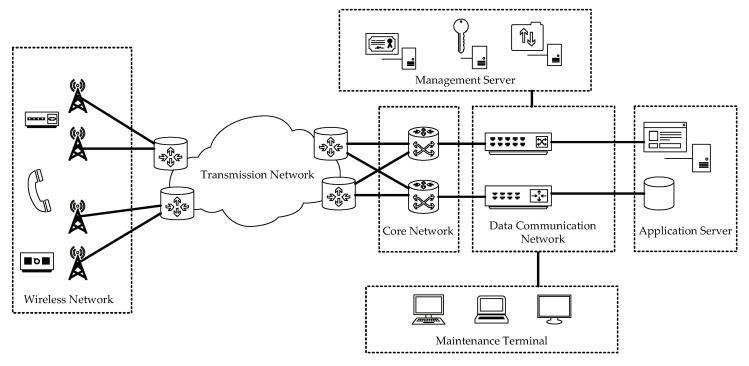
A topology diagrammatic sketch of a railway company communication network.

Railway companies often have a series of sites geographically separated down the railway line. To connect these sites to their service center, a high-quality channel for transferring data is necessary. An optical transport network provides the capacity to schedule and transmit various types of businesses with different particle sizes. In general, it consists of a long-distance physical optical fiber cable and a large amount of switching equipment. Faults often arise in these components. In this study, the structure of the transmission network is a synchronous digital hierarchy (SDH) network. The railway line is approximately 628 km long, and there are 34 sites distributed along the railway line. Figure 5 shows a topology diagrammatic sketch of the communication network of the railway company.

As the backbone of the communication network, transmission networks carry a large number of important services for train running, such as synchronous control for both locomotives and trains. Radio train dispatching communication controls the train tail device, sends and receives dispatching instructions to and from running trains, and identifies and checks the numbers of running trains, which are sensitive to network communication server quality. More importantly, those services are the basis for organizing railway transport, enhancing production efficiency, and protecting railway operation safety. Continuous monitoring of the performance of a transmission network and localizing root causes quickly and accurately after faults occur are of crucial importance for network managers to ensure the reliability and quality of the communication network. Any unexpected or prolonged downtime that leads to transportation interruption largely decreases the loyalty of customers and drastically affects the efficiency of transport.

Alarms are our main information source for understanding the operating states of the equipment and performing fault localizing tasks in communication networks. In this case study, the alarm data were obtained from the alarm management system via the data interface. The fault information was obtained from the equipment specifications provided by the equipment vendors. There are several alarm attributes in every alarm message. Based on our approach, both alarm name and alarm position were selected for our case study.

**Figure 6:**
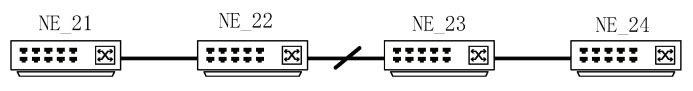
A fault scenario in the transmission network. We assume a break in the fiber link between NE_22 and NE_23.

Figure 6 depicts a fault scenario consisting of four pieces of equipment; namely, NE-21, NE-22, NE-23, and NE-24, geographically separated over four sites, respectively. Here, NE refers to network entity. The number following the NE represents the geographical position code of the network entity. We assume that the fiber link between NE-22 and NE-23 breaks. NE-22 is positioned upstream of NE-23, and the business data then flow from NE-22 to NE-23. The broken link leads to a communication break between NE-22 and NE-23. As a consequence, the sites near NE-23 may experience an abnormal condition and report a large number of alarms. For example, NE-23 may trigger the R-LOS (Receive Loss Of Signal) alarm due to the broken link. The multiplex section protection mechanism of the SDH network was started up simultaneously and changeover occurs. The MS-APS-INDI-EX (Multiplex Section-Protect switch indicate expand) and APS-INDI (Automatic Protection Switching State indicate) alarms at NE-23 were then reported. We also received R-LOS and ALM-GFP-dLFD (Generic Framing Procedure Loss of frame delineation) alarms at NE-22 and NE-23, respectively, due to the loss of signal and the loss of frame alignment.

The reported NE_22, NE_23 and NE_24 alarms are shown in Table 3. An experienced operator may speculate the probable causes of these alarms: (1) a fault in the single board of an optical switch in NE_22, (2) a fault in the single board of an optical switch in NE_23, and (3) a broken or degraded link between the optical fiber and NE_22 or NE_23. These scenarios may cause a loss of time due to troubleshooting and may evolve into catastrophe events.

**Figure 7:**
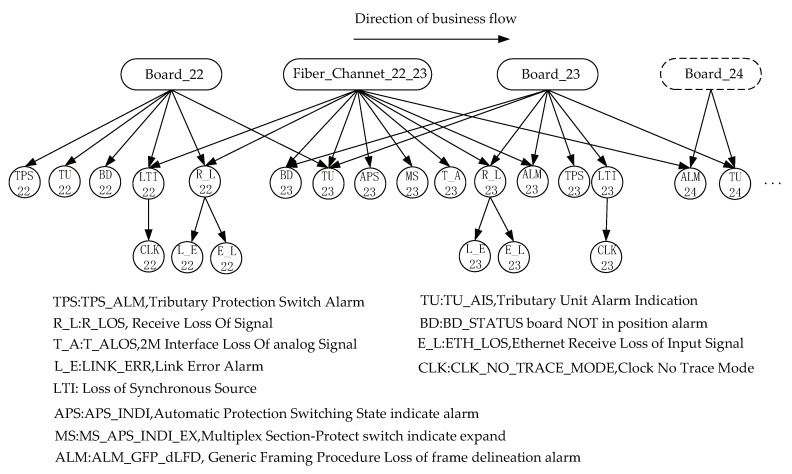
A polytree belief network with two scenarios: a break in the fiber link and a board fault. The number following the alarm and fault identifier represent the the network entity code in which events occur. The dotted line box represents the non-neighbor node of fault node.

In order to find the root cause among the three most probable causes with minimal time, we combined the human expertise and the knowledge learned from the historical alarm log in the belief network, as shown in Figure 7.

In Figure 7, all of these alarms are mapped to the corresponding nodes in the belief network. Let us take these alarms as evidence in the causal reasoning. The fault localization process then starts from these alarm nodes. Based on the previously proposed inference algorithm, we find the right root cause to be consistent with the initial consideration in the shortest possible time. After three iterations, the probability distributions of the three potential causes are p(Board_22)=(0.0131,0.9869),p(Fiber_Channal_22_23)=(0.9999,0.0001) and p(Board_23)=(0.1410,0.8589), respectively. It is evident that Fiber_Channal_22_23 is the fault root cause of these alarms, and we only require less than 0.0006 seconds.

**Figure 8:**
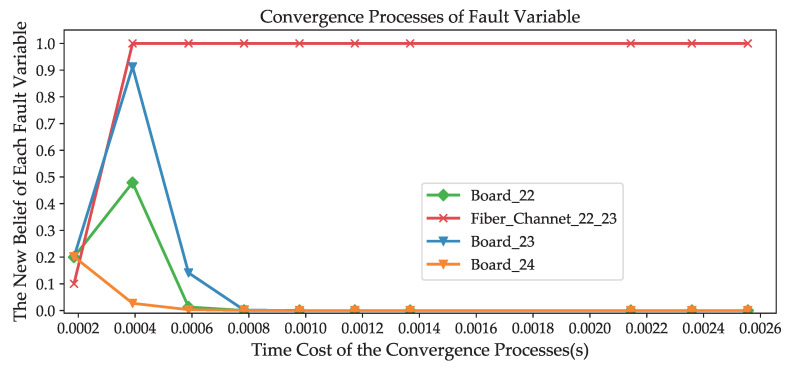
The convergence processes of fault variables.

The message exchange in the inference process is a dynamic iterative process; nevertheless, the final belief distribution of each node will converge with its own unique equilibrium state. Figure 8 depicts the dynamic convergence process of each variable.

## 6. Evaluation and Discussion

In this section, we present a series of experimental simulations to assess the performances of the proposed fault localization techniques according to four metrics: convergence speed, reliability, the ability to deal with multiple-source faults, and the capability of identifying faults in an uncertain environment.

### 6.1. Evaluation Methodology

#### 6.1.1. Generation of the Belief Network

Based on the topology of the transmission network and the dependency relationship of their entities, a belief network was built combining human expertise and knowledge learned from observed data. Building a belief network structure and estimating conditional probability parameters are other important works, but these are beyond the scope of this study.

#### 6.1.2. Experiment Settings

The fault scenarios and experimental data come from fault analysis reports. Here, 112 fault analysis reports from February 2016 to August 2019 were gathered. Each report records a large number of alarms and faults inferred from these alarms. In order to estimate the performance of the proposed approach, we selected 10 reports from the 112 fault analysis reports. In these 10 reports, we randomly added some noise alarm data.

In each experiment, one report is selected, and all the alarms in the report are viewed as symptoms. Their status values are set as 1. The fault localization process is then started from these alarms. According to the requirements of different evaluation indexes, corresponding evaluation results are obtained.

In addition, the data used for the experiments using support vector machine (SVM) and multi-layer perceptron (MLP) approaches are generated using a simulation environment (Simulation Laboratory). For example, we trained the MLP model with 1000 data samples for link failures, and the test data set includes 150 data samples. SVM and MLP are classic data analysis methods for the classification problem, and have been widely used in the literature for fault identification and localization [25,26,49,50].

### 6.2. Evaluation Result

#### 6.2.1. Convergence speed

The convergence speed is an important metric for assessing the validity of a good dynamical system. In our model, alarms are viewed as a perturbation that, through the network between neighboring nodes, is the driving force of message propagation and fault inferences. The network reaches a new equilibrium through several iterations.

Table 4 shows the experimental results obtained for networks with sizes ranging from 100 to 2000 nodes. These results show that the message propagation approach has a good fault localization performance in terms of convergence speed. As the size of the network increases, there is very little increase in the time required to reach equilibrium states. For example, the simulation results show that the approach requires 0.0054 s to reach the equilibrium state in a 100-node network, while reaching the equilibrium state for a 2000-node network only requires 0.0868 s.

The faster the localization of the fault’s root cause, the less substantial the impact of the fault on the network. In Table 5, we present the time taken using the different approaches to localize the fault’s root cause in the network. The experiments were carried out in a belief network with 2000 nodes. Note that the localization times of the SVM and MLP approaches are not affected by the sizes of networks.

Based on the experimental results, the PTNORgate approach significantly reduces the localization time of the BN. SVM and MLP approaches do not perform well in terms of time. This is because they require a long training period to train their learning models.

#### 6.2.2. Reliability

Reliability is an important metric for assessing a fault localization system. It is used to measure the trustworthiness of a system’s judgments. We will use the following metrics to estimate the reliability of the proposed approach.

- Precision: The ratio of the number of fault analysis reports correctly identified over the total number of fault analysis reports identifying faults. The higher the value of precision, the lower the misdiagnosis rate, and vice versa. The precision value can be computed as follows:
  (6) $$P=\frac{T_{P}}{T_{P}+F_{P}}$$
  where *P* is the precision value, $T_{P}$ is the number of true positives, and $F_{P}$ is the number of false positives.
- Recall: The ratio of the number of fault analysis reports correctly identified over the number of fault analysis reports that actually occurred. The higher the value of recall, the lower the misdiagnosis rate, and vice versa. The recall value is computed as follows:
  (7) $$R=\frac{T_{P}}{T_{P}+F_{N}}$$
  where $F_{N}$ is the number of false negatives.
- $F_{1}$-Score: $F_{1}$-Score is the harmonic average of the precision and recall. Higher the value of $F_{1}$-Score, the better the performance of the approach. The $F_{1}$-Score value can be computed as follows:
  (8) $$F_{1}-Score=\frac{2PR}{P+R}$$

**Figure 9:**
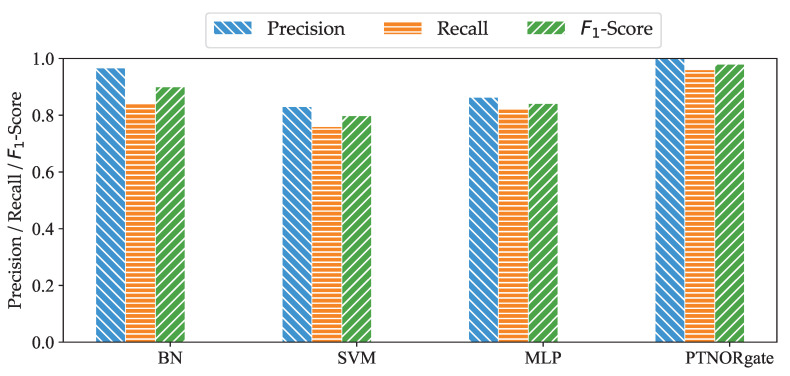
Comparison of reliability obtained with different fault localization approaches.

In Figure 9, we plot the precision, recall, and $F_{1}$-Score values of the various approaches. The results show that the PTNORgate approach achieves 100% precision, closely followed by BN with a precision of 96.63%. This indicates that the cause–effect inference is suitable for fault localization. The results also show that the PTNORgate approach localizes fault with minimal misdiagnosis. We obtained a recall of 96.07% for PTNORgate. This high recall value implies that PTNORgate has a low false negative rate. MLP attained only 86.3%, 82.1% and 84.15% for precision, recall, and $F_{1}$-Score, respectively. This may be due to the overfitting problem. SVM has the worst performance among the four approaches; nevertheless, it achieved 83.06% precision, 76% recall, and 79.81% $F_{1}$-Score. Among the four approaches, the PTNORgate approach clearly outperforms others. This shows the reliability of the PTNORgate approach in fault localization.

#### 6.2.3. Capability to Deal with Multi Source Fault

We have demonstrated that the proposed approach has a good performance in terms of convergence speed and reliability when dealing with a single fault. Now, we evaluate the capability of the approach to deal with multiple, simultaneous faults. A 2000-node network and 112 fault analysis reports are used.

The test process is as follows: Two fault analysis reports were randomly selected as the fault scenario of the test. The fault localization model was then run and the root cause was determined. Whether the diagnosis results were consistent with the fault analysis report was checked. Another two reports were selected from the remainder of the fault analysis reports. The entire procedure was repeated until all fault analysis reports were tested. The results show that this method can optimally solve the problem of fault localization in multiple fault scenarios.

**Figure 10:**
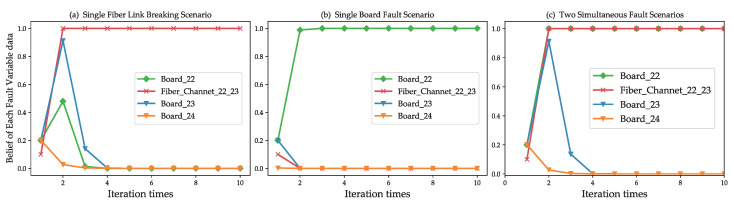
Fault localize in multiple fault scenarios. (**a**) A fiber link break fault, Fiber_22_23 obtained the highest fault probability. (**b**) A function board failure, Board_22, was localized. (**c**) When the fiber link break (Fiber_Channet_22_23) and function board failure (Board_22) occurred simultaneously, the two of them were identified.

Taking the failure scenarios described in Section 5 as examples, Figure 10a,b show the iteration processes and localization results of a single fault scenario, such as a fiber link break or a function board fault. Figure 10c shows the iteration processes and localization results of two faults occurring simultaneously. The results show that the method can accurately identify fiber link faults and function board faults.

#### 6.2.4. The Ability to Identify Faults in Uncertain Environments

A communication network is a complex and dynamic system. Fault localization approaches need to be able to deal with the uncertainty of a network.

**Figure 11:**
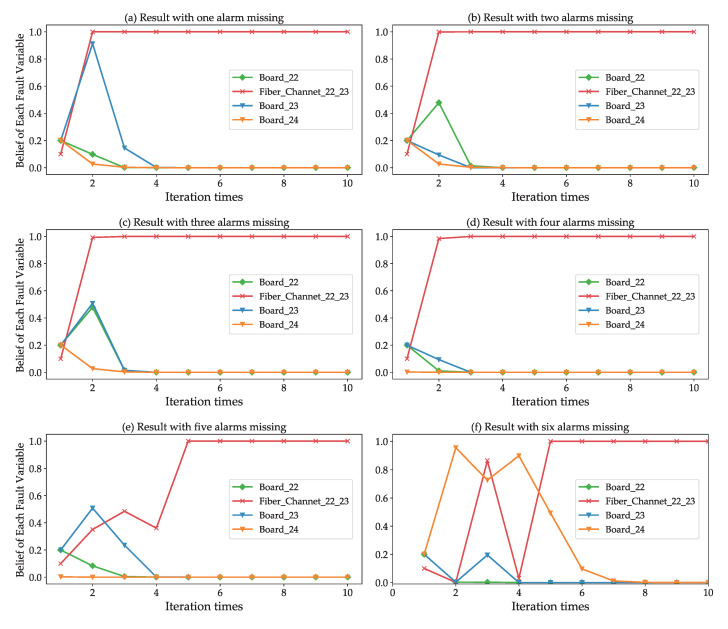
Fault inference in uncertainty environments. There are a total of twelve alarms in the fault scenario of the broken link between NE-22 and NE-23. (**a**–**f**) show the iterative process and results of fault location with 1 to 6 alarms missing, respectively.

We took the fault scenario described in Section 5 as an example to consider fault inferences under uncertain conditions. Figure 11a–f show the iteration processes and results where one to six alarms are removed from the alarm lists. The alarms were removed randomly during the experiment. We received a total of 12 alarms in this fault scenario. Although the reasoning process was hard, our approach still identified the root cause of the fault when six alarms were removed.

**Figure 12:**
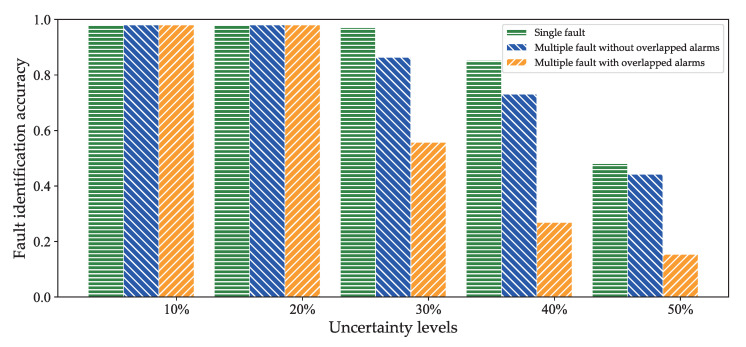
Fault identification accuracy under different uncertainty levels.

Figure 12 shows the fault identification accuracy at different levels of uncertainty. Considering that the uncertainty levels that exceed 50% are implausible in real-life fault scenarios, and that it is impossible to perform an effective fault inference, we used five configurations to generate different uncertainty levels: 10% of alarms are missing, 20% of alarms are missing, 30% of alarms are missing, 40% of alarms are missing, and 50% of alarms are missing. In Figure 12, it is apparent that uncertainty levels below 20% barely influence the fault localization results. The fault identification accuracy decreases as uncertainty levels increase. When considering multiple, simultaneous fault scenarios, we observe that the fault identification accuracy of multiple faults without overlapping alarms is higher than that of the multiple faults with overlapping alarms at the same uncertainty levels (except for 10% and 20%). This phenomenon is consistent with the local operation of the polytree structure.

## 7. Conclusions and Future Work

In this paper, we propose a framework for fault localization in communication networks. The clear structure of the data storage, inference, and message transmission in the overall framework exposes information about the fault inference procedure, and facilitates the development of a message propagation approach that is applicable to various fault localization problems. Fault localization in an event-driven manner improves the degree of the automation of fault localization and reduces human intervention in the fault localization process. The PTNORgate model was used to reduce the computational complexity of the inference process.

An extensive assessment of our proposed approach was carried out in experiments and shows its benefits in comparison to other approaches. These results show that our approach provides an efficient framework for root cause localization in terms of convergence speed, reliability, automation, and the ability to deal with multiple-source faults under uncertain environments. On the contrary, SVM and MLP performed poorly in our work due to multiple fault classifications and overfitting problems, respectively.

In the future, we plan to further investigate the accuracy of the dependency relationship between failures and alarms in the networks. Indeed, the reliability of a fault localization model requires an accurate dependency relationship between variables. Discovering and identifying dependency relationships between failures and alarms are complex tasks for a large-scale network. Therefore, the next step is to propose a method that automatically learns the causal relationship among failures and alarms purely from the data.

## Figures and Tables

**Table 1:** Network parameter information of each node in the network parameter table.

Relationship	Name	Prior Probability	Belief	$q_{i}$	$\pi_{x}^{0}\left(u_{i}\right)$	$\lambda_{Y_{i}}^{0}\left(x\right)$	$\lambda_{Y_{i}}^{1}\left(x\right)$
Self	*X*	p(x)	bel(x)		$\pi^{0}\left(x\right)$	$\lambda^{0}\left(x\right)$	$\lambda^{1}\left(x\right)$
father	$U_{1}$			$q_{1}$	$\pi_{x}^{0}\left(u_{1}\right)$		
father	$U_{i}$			$q_{i}$	$\pi_{x}^{0}\left(u_{i}\right)$		
⋮	⋮			⋮	⋮		
father	$U_{m}$			$q_{m}$	$\pi_{x}^{0}\left(u_{m}\right)$		
child	$Y_{1}$					$\lambda_{Y_{1}}^{0}\left(x\right)$	$\lambda_{Y_{1}}^{1}\left(x\right)$
child	$Y_{i}$					$\lambda_{Y_{i}}^{0}\left(x\right)$	$\lambda_{Y_{i}}^{1}\left(x\right)$
⋮	⋮			⋮	⋮		
child	$Y_{n}$					$\lambda_{Y_{n}}^{0}\left(x\right)$	$\lambda_{Y_{n}}^{1}\left(x\right)$

**Table 2:** The final belief distribution of each variable.

Variable	Fault 1	Fault 2	Fault 3	Alarm 1	Alarm 2	Alarm 3	Alarm 4	Alarm 5
Value	(0.1418,	(0.1882,	(0.9346,	(0.16,	(0.9078,	(1,0)	(1,0)	(1,0)
	0.8582)	0.8118)	0.0654)	0.84)	0.0922)			

**Table 3:** Alarm data gathered from NE-22, NE-23 and NE-24.

Sites	NE-22	NE-23	NE-24
Alarm	R-L, LTI, CLK	BD, TU, APS, MS, T-A, R-L, ALM, E-L	ALM

The detailed description of the alarm identifier is shown in Figure 6.

**Table 4:** Time required for each iteration in different scales networks.

Number of Nodes	100	200	400	600	800	1000	2000
	0.0005	0.0010	0.0020	0.0028	0.0042	0.0053	0.0097
	0.0006	0.0010	0.0026	0.0038	0.0042	0.0050	0.0094
	0.0006	0.0011	0.0020	0.0028	0.0040	0.0050	0.0097
	0.0006	0.0010	0.0020	0.0035	0.0041	0.0053	0.0101
Time per iteration (s)	0.0007	0.0010	0.0020	0.0029	0.0043	0.0054	0.0097
	0.0006	0.0010	0.0022	0.0034	0.0041	0.0050	0.0093
	0.0006	0.0010	0.0020	0.0029	0.0045	0.0051	0.0094
	0.0006	0.0010	0.0020	0.0033	0.0040	0.0053	0.0096
	0.0006	0.0010	0.0023	0.0030	0.0051	0.0053	0.0099
Time to reach equilibrium (s)	0.0054	0.0091	0.0191	0.0284	0.0385	0.0467	0.0868

**Table 5:** Comparison of times required for localization of fault in a 2000-node network.

	Localization Methods	Time Required for 2000 Nodes (s)
	Traditional Bayesian network (BN)	6.3207
	Support vector machine (SVM)	2.0763
	Multi-layer perceptron (MLP)	0.2498
	Polytrees with noisy OR-gate (PTNORgate)	0.0868
